# Automation system for neutron activation analysis at the reactor IBR-2, Frank Laboratory of Neutron Physics, Joint Institute for Nuclear Research, Dubna, Russia

**DOI:** 10.1007/s10967-016-4864-8

**Published:** 2016-05-13

**Authors:** Sergey S. Pavlov, Andrey Yu. Dmitriev, Marina V. Frontasyeva

**Affiliations:** Frank Laboratory of Neutron Physics, Division of Neutron Physics, Department of Neutron Activation Analysis and Applied Research, Joint Institute for Nuclear Research, Str. Joliot-Curie, 141980 Dubna, Moscow Region Russia

**Keywords:** Reactor neutron activation analysis, Automation of neutron activation analysis, Sample changers, Software for automation of neutron activation analysis

## Abstract

The present status of development of software packages and equipment designed for automation of NAA at the reactor IBR-2 of FLNP, JINR, Dubna, RF, is described. The NAA database, construction of sample changers and software for automation of spectra measurement and calculation of concentrations are presented. Automation of QC procedures is integrated in the software developed. Details of the design are shown.

## Introduction

In Joint Institute for Nuclear Research neutron activation analysis is carried out using the installation REGATA at the IBR-2 reactor of Frank Laboratory of Neutron Physics [1]. The IBR-2 reactor is a fast neutron pulsed reactor with narrow neutron pulse (240 µs) and small repetition rate of pulses (5 Hz). The reactor staff permanently performs experiments with different neutron moderators in order to increase the thermal neutron flux. That is why the relative method of NAA is used.

In the framework of international programs in life sciences and materials science at this facility, analyses of a large number of samples are carried out. In such circumstances automation of analytical procedures, data processing, registration of samples and interexchange of information is of high priority. This requires organization of labeling, storage and recording of analyzed samples as well as certified reference materials and flux monitors, irradiations, measurements and processing of *γ*-spectra of induced activity, and systematization of analytical results.

Improving of the quality and productivity of NAA can be achieved through:

- storage of information about all stages of NAA in electronic database;
- automation of data input for spectra analysis;
- automation of sample preparation and irradiation of samples, CRMs and flux monitors;
- automation of measurements of spectra using sample changers;
- automation of the process of calculating the concentrations, storage of analytical results, and data reporting;
- use of programmable QC procedures, rapid statistical analysis of the results;
- fast check of all stages of analysis and search for any information on NAA from any computer in the department.

This paper describes how these requirements have been implemented by development of new hardware and software for comprehensive automation of NAA at the IBR-2 reactor.

## Software for automation of NAA

The software package for automation of neutron activation analysis at IBR-2 reactor in Dubna includes the following programs [2, 3]:

1. The database of information about all steps of analysis.
2. A set of service programs to automate and facilitate the completion of the database:
  Environment of NAA
  Information about clients
  Information about samples
  Weight of each sample
  Journal of measurements
  Search for most appropriate CRM
3. A program for automation of measurement of the induced activity using the program for spectra analysis Genie-2000 and Batch Support Tools S561 Genie-2000 (Canberra).
4. A program for calculation of element concentrations based on analysis of gamma spectra with the program Genie-2000.

**Fig. 1:**
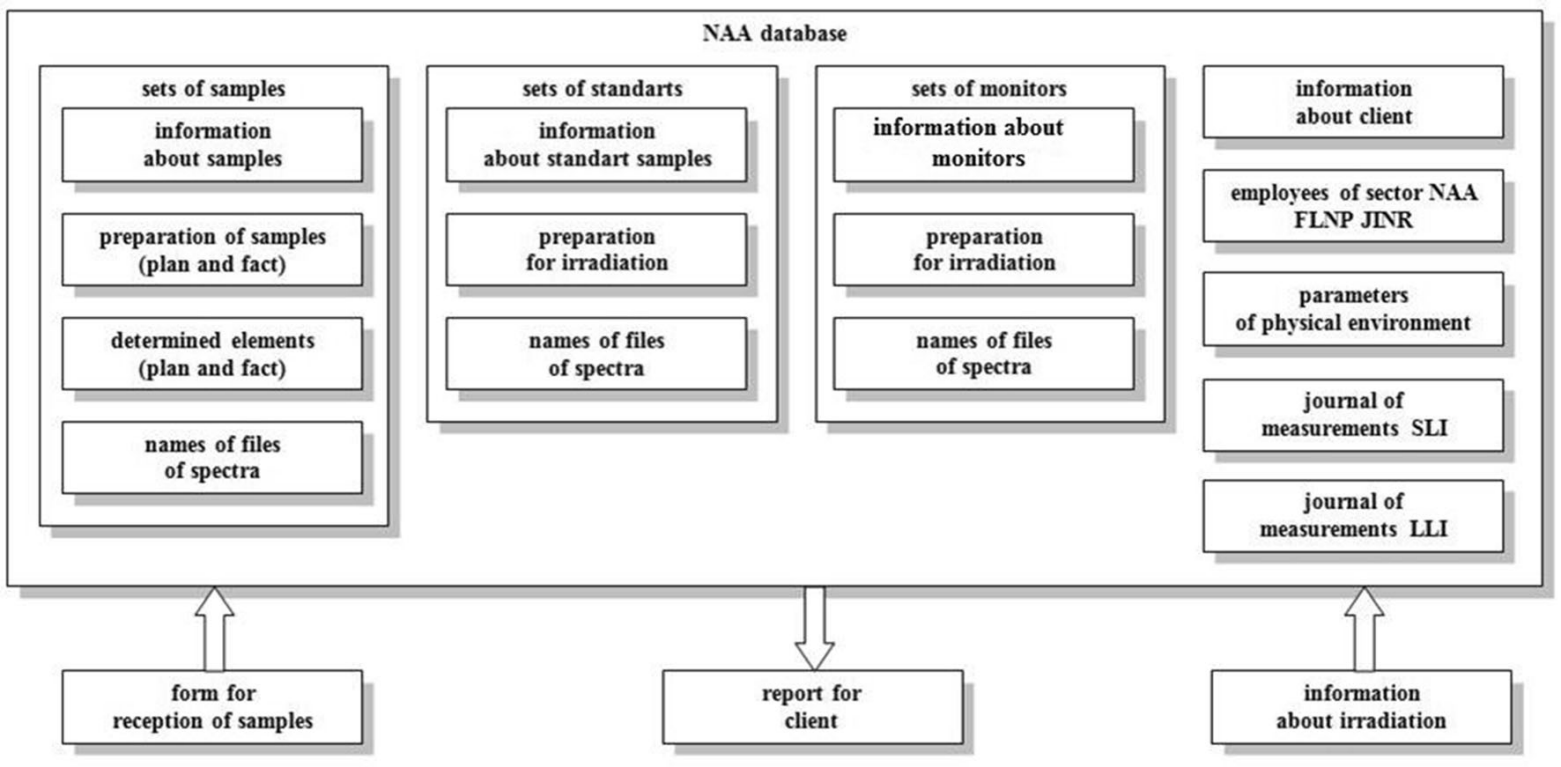
The structure of the database

All information about clients, samples, CRMs, flux monitors and about all steps of NAA at the IBR-2 reactor is stored in a special software—NAA database. The database diagram is presented in Fig. 1.

The NAA database was developed using the MS SQL-server software. It is stored at hard disc of server with a copy at a second hard disc. Once per week all information is copied to the external hard disc. The access to the database is arranged through the net and is possible with three levels of access, using individual passwords:

- lowest level is for searching and receiving any information about NAA;
- middle level allows to change and save information in same parts of database;
- highest level gives opportunity to change and save any information.

The creation of such databases gives the opportunity for electronic circulation of documents, which is very useful taking into account a large distance between offices and reactor. The search and entering of any information in the database is very fast and comfortable. Besides this, database allows carrying out statistical analysis of the obtained results, which is particularly useful in analysis of environmental samples. All information saved in the database is divided into several parts: information about samples, CRMs and flux monitors, beginning from their receiving or purchasing till the results of analysis; information about irradiation; auxiliary information about clients; parameters of physical environment and statistical analysis.

**Fig. 2:**
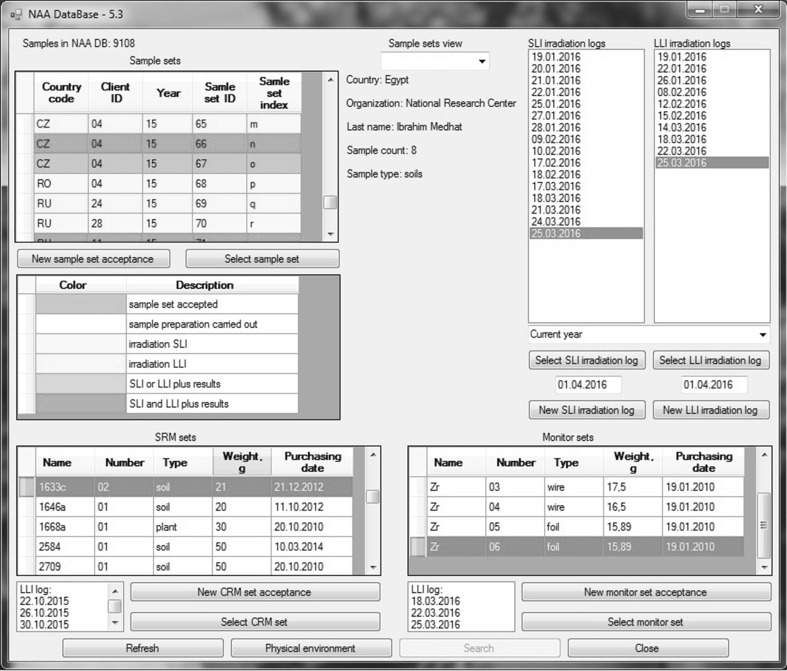
The main window of the database

Figure 2 shows the main window of the database interface. It has many additional windows, by means of which one can insert and check information about customers and received samples, used CRMs and neutron flux monitors and their residual amount, as well as data on all steps of the NAA: receipt of samples, sample preparation, irradiation, spectra measurement and processing, and statistical analysis of the results. Each sample set in the left top corner of the window has its own unique code, colored according of fulfilled steps of analysis. The opened log of irradiation in the right top corner contains all information about short or long irradiation. In the lower part of this window it is possible to find any information about CRMs and flux monitors.

**Fig. 3:**
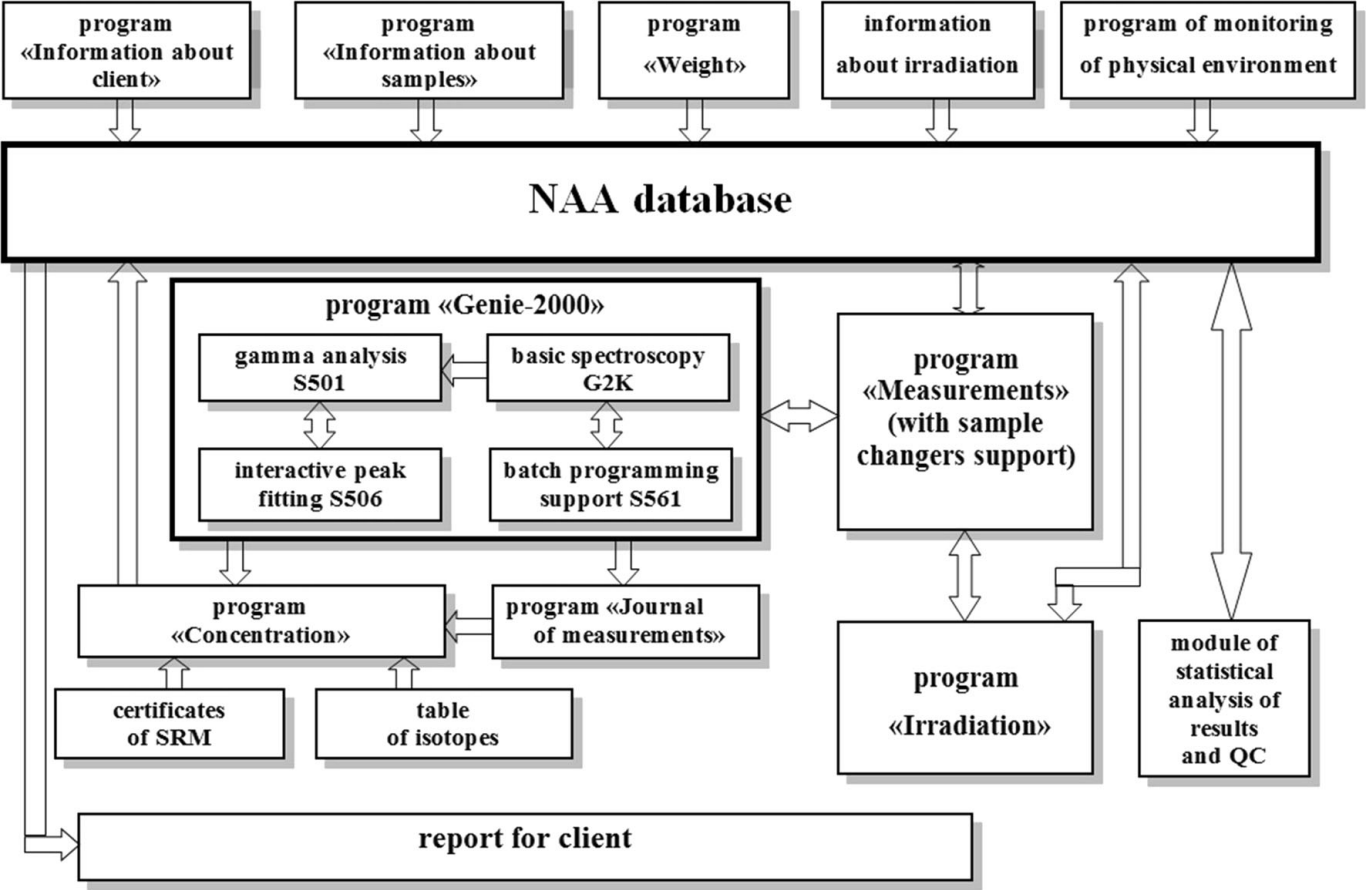
The software structure for automation of NAA at IBR-2 reactor

Most of the information is entered into the database automatically using a set of service programs. Part of information is entered into the database manually. It is very comfortable and fast. In the future, this part of information will be reduced with further development of software and setup for irradiation at the IBR-2 reactor. The structure of software for automation of NAA is shown in Fig. 3.

In addition to the database several service programs were developed for automation of entering information in the database. These are mentioned above: *Environment of NAA*, *Information about clients*, *Information about samples*, *Weight*, *Journal of measurements* and *Standard search.*

The program *Environment of NAA* allows running the database and some other programs, opening the journals for registration of discrepancy in NAA and the time of filling the detectors with liquid nitrogen. Using this program, it is possible to send message by e-mail and directly to the screens of computers of all employees of our department.

The program *Weight* automatically saves the weights of samples, CRMs and flux monitors, prepared for short and long irradiation, directly from balance in the database.

The program *Journal of measurements* reads the information from the headers of measured spectra and compiles the list of these spectra. The review of this list allows determination of missing or wrong spectra (with high dead time, short measurement time) which must be re-measured.

Using the program *Standard search* it is possible very fast to find the most suitable CRM with certified values of concentrations and lowest values of uncertainty for any element or set of elements. This program can show all certified concentrations of elements in one or several chosen reference materials, too.

## Automation system for measurement of *γ*-ray spectra of induced activity

Four spectrometers are used for *γ*-spectra measurement. Three of them are equipped with sample changers. Each sample changer consists of a two-axis linear positioning module M202A by DriveSet (DriveSet.de, Germany) company and a disk with 45 slots for containers with samples manufactured in JINR workshops [3].

**Fig. 4:**
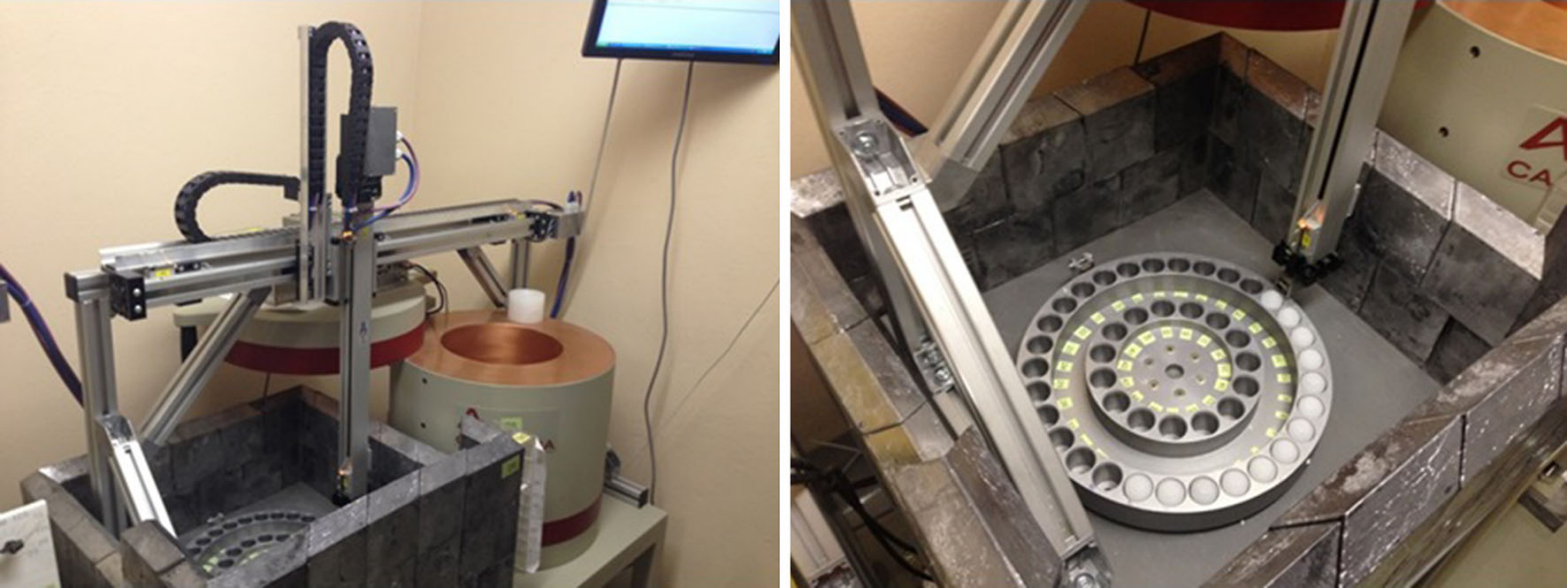
General view of the sample changer

Each module M202A is fixed above two metal tables with adjustable feet by means of the Bosch Rexroth aluminum module profile system. On one of the tables, a rotating disk with samples is installed. The dewar with the detector is placed below another table, and the head of detector is above the surface of the table. The disk with samples, as well as the detector, are surrounded with a shielding (Fig. 4).

The disk is rotated by a stepper motor EPL64/2 with a two-way shaft from Nanotec (nanotech.com) company. On one side, the shaft of motor is connected with the shaft of the disk by means of a coupling. An incremental encoder is mounted on the other side of motor shaft. The initial cell in the disk is determined by an electromagnetic sensor while an increment encoder controls the selection of any other cell with samples on the disc. Positioning accuracy of the disc can reach 0.01°.

**Fig. 5:**
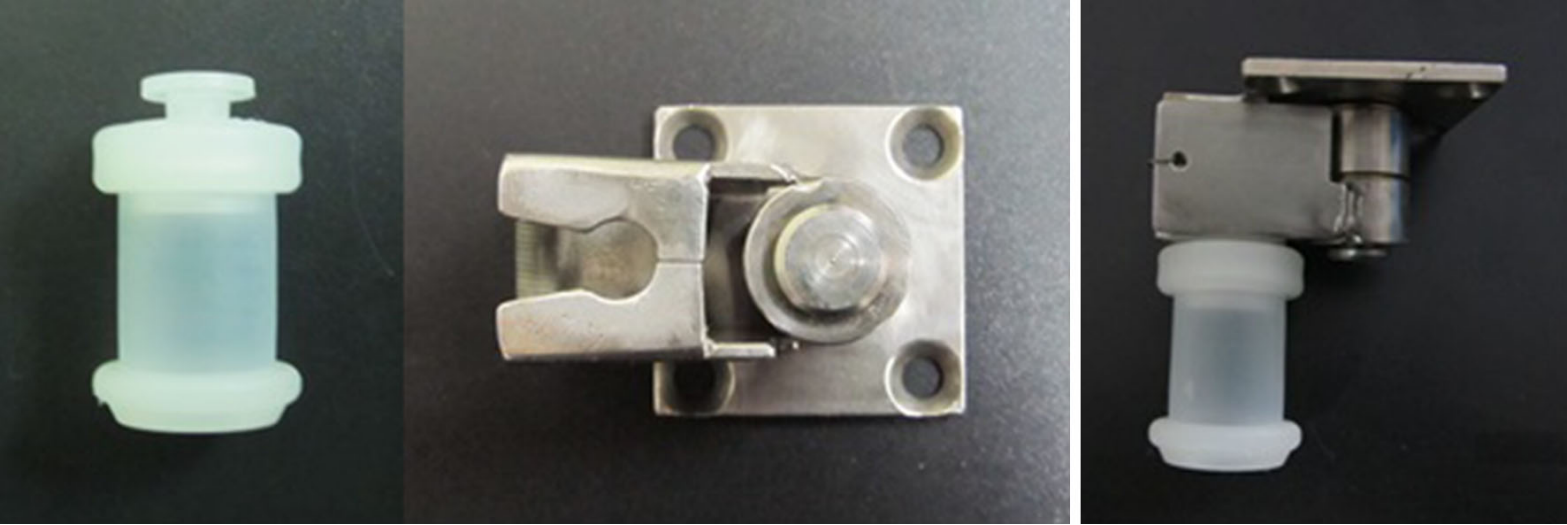
The grab of container

The movement of containers from the disk to the detector and back is carried out by a device M202A manufactured by DriveSet company. It comprises horizontal and vertical linear positioning modules. Each axis is made of high-strength aluminum profile with integrated hardened steel rods. A carriage with precision guide rollers moves along the track. The carriage of each module moves by means of a screw with trapezoidal thread, which is rotated by a stepper motor. The brake for the vertical axis is not needed because of the self-locking trapezoidal thread. Each axis is provided with two ends and one reference sensor as well as with a linear incremental encoder, which allows the determination of the position of the carriage. The end sensors exclude the possibility of damage of the devices while moving to the physical boundaries of the axes. Reference sensors allow one to specify the initial positions of the modules. Positioning accuracy can reach 0.1 mm. Maximum speed of movement along both axes is 0.08 m/s and acceleration upto 1 m/s^2^ with a maximum load of 1 kg. The maximum vertical movement for this module is 400 mm and for the horizontal one 800 mm. Harnesses are laid into movable cable-channels. The special spring-pressed grab is used to capture the container from the disk (Fig. 5).

Control of linear positioning module M202A and disc with samples is provided by a Xemo S360U controller by Systec (systec.de) company. Each sample changer uses its own controller. Each controller can control up to four axes. All controllers are connected to the same PC via USB ports. The libraries for OS Windows, necessary for developing the program to control the devices and measurements of spectra were included in the purchased package.

**Fig. 6:**
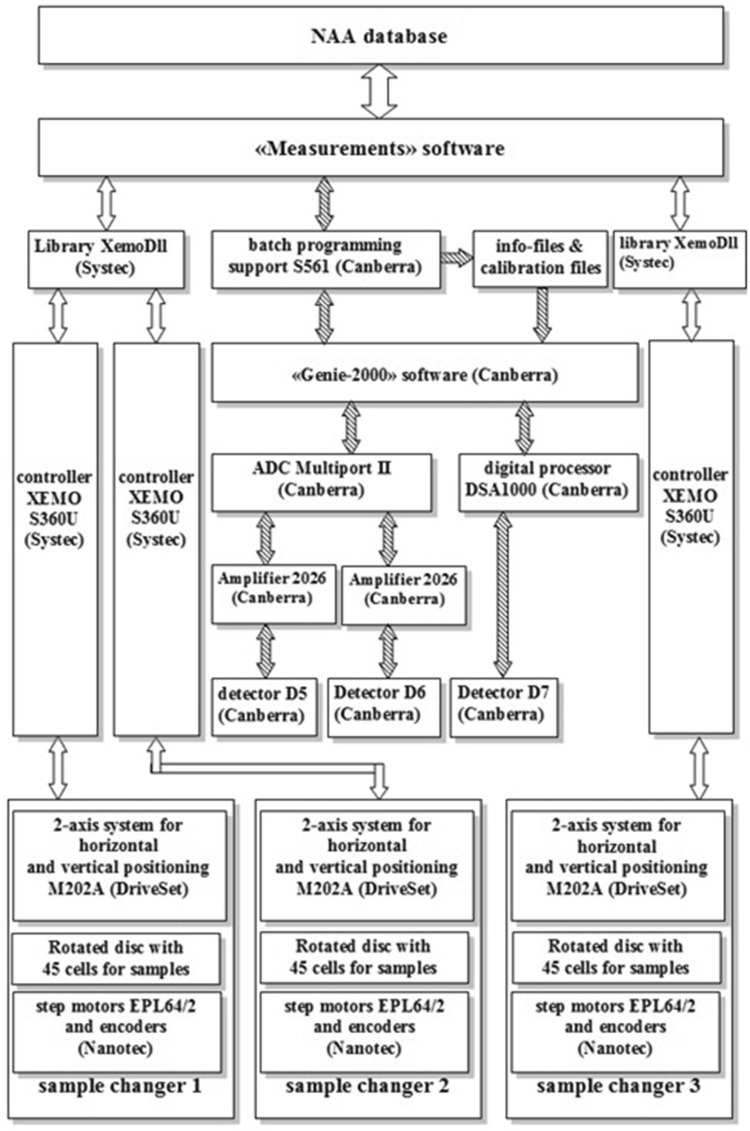
The block diagram of the automation system for γ-spectra measurement

Each container selected from the disk is moved to the detector and is held above it during the measurement of the spectrum. The spectra are measured in one of the four fixed height positions above the detector. Control of the container position during the measurement of the spectra is performed by incremental encoders. After completion of the measurement the container is returned to the same location on the disk which then rotates for selection of the next sample. A block diagram of the automation system for measurement of gamma-ray spectra is shown in Fig. 6.

The program *Measurements* was developed to automate measurements of *γ*-spectra of induced activity and to control the sample changers of four spectrometers during measurements simultaneously.

The program is written in Visual Basic. The software Genie-2000 (Canberra) is used for spectra measurements. The XemoDll library by Systec company was used to create a program module to control the sample changers. Interaction with the program Genie-2000 is performed by means of a dynamically generated program using the REXX programming language (REstructured eXtended eXecutor—“restructured extended executor”) in the Batch Support Tools S561 for Genie-2000. The structured query language (SQL) is used to work with the database.

**Fig. 7:**
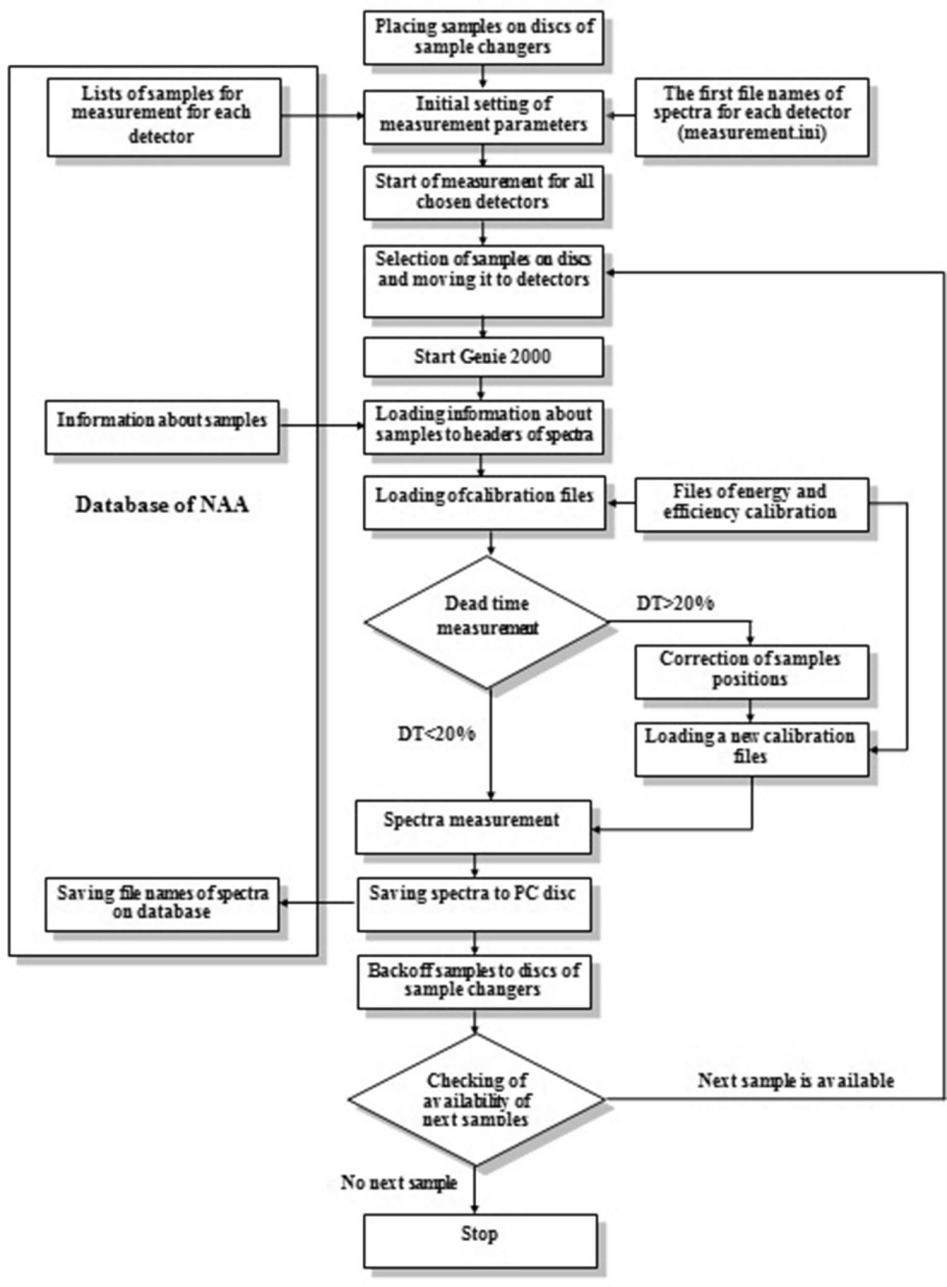
The logic diagram of the program *Measurement*

The logic diagram of the program *Measurement* is shown in Fig. 7.

**Fig. 8:**
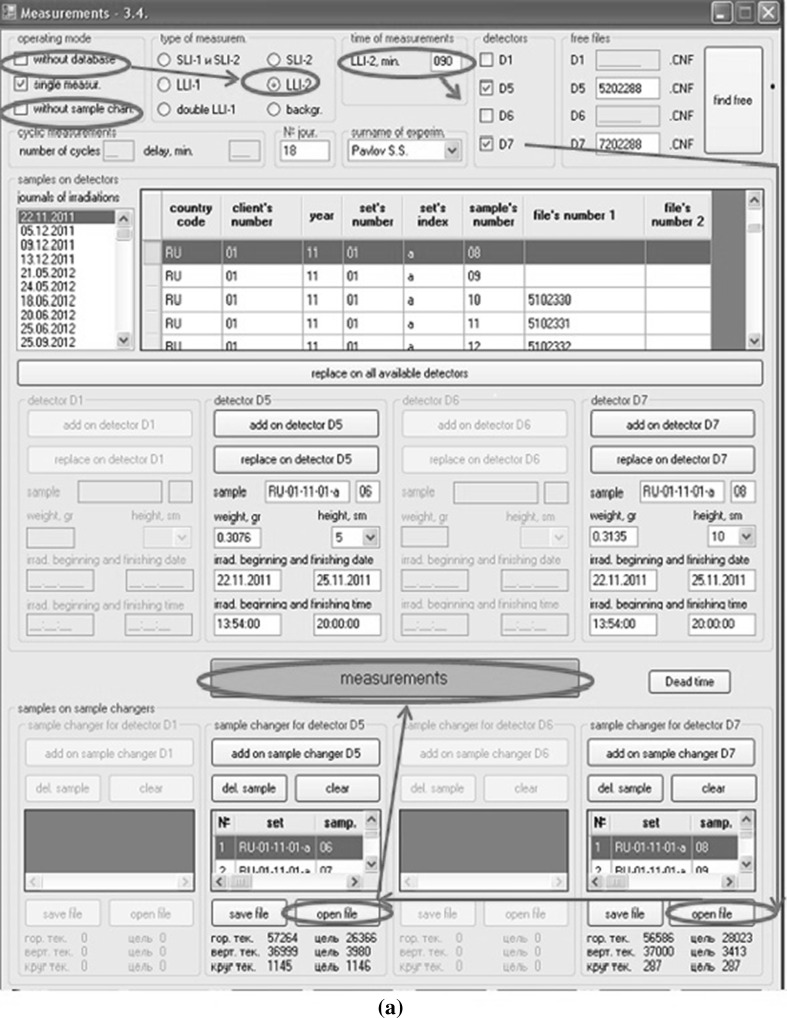

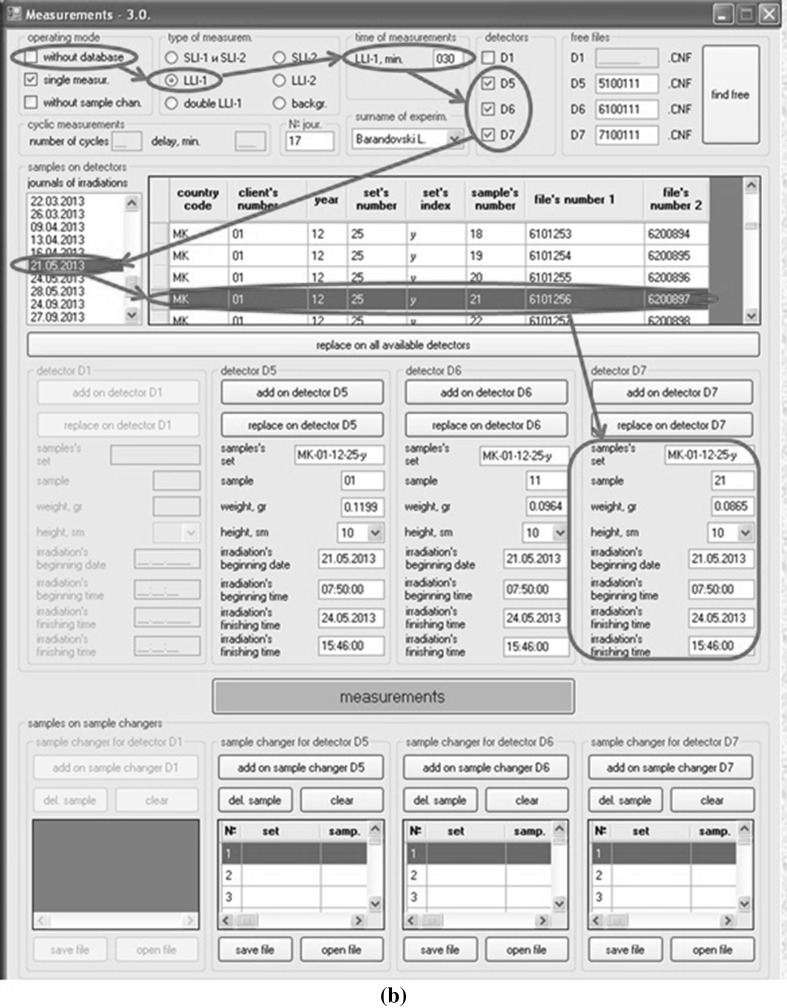
**a**, **b** Windows of the program for automation of spectra measurement in two different operation modes: with and without sample changers

An installation of samples to a disc and spectra measurement are carried out according the list of measured samples received automatically from the database. After start of the program one must make the initial settings. The sequences of two possible settings for measurements with database and with sample changers (a) and with database but without sample changers (b) are shown by arrows on Fig. 8a, b accordingly. The program automatically proposes the next free names of spectra for each detector. The format of name of spectrum is AB12345, where *A* is detector number, *B* = 0, 1, 2 is type of spectrum (0 is spectrum of short-lived isotopes, 1 is spectrum of medium-lived isotopes and 2 is spectrum of long-lived isotopes) and 12345 is the sequence number. Such names of spectra are necessary for proper operation of the program for calculation of element concentrations.

After the beginning of spectra measurements according to the name of sample all the necessary data for calculation of concentrations are taken from the database and from the corresponding files and recorded automatically in the fields of the program and in the headers of spectra. It is necessary to notice that Genie-2000 at first makes the test measurement of spectra for each detector during 10 s to determine the dead time. If dead time is more than 20 %, the program changes the position of samples on detectors. After the end of spectra measurements, the program stores the spectra to hard disc of PC and file names of measured spectra to the database. Then measured samples are returned to the same cells on the disc and the samples from the next cells are moved to the detectors. Maximum number of spectrometers and sample changers controlled by this program is four.

Each sample changer is equipped by Ethernet video camera, which allows controlling the operation of the system in remote mode, to make a picture of the sample during measurement and save this picture in the database. Thereby possible human mistakes during installation of samples to the discs can be revealed.

In the case of fault, a special device (Netping 4/PWR-220 v3/SMS) can send SMS about of interruption of measurements and reload the controllers of sample changers and computer in remote mode.

The program allows minimizing human involvement in routine long-term measurements of the spectra of the induced activity.

## Program *Concentration*

**Fig. 9:**
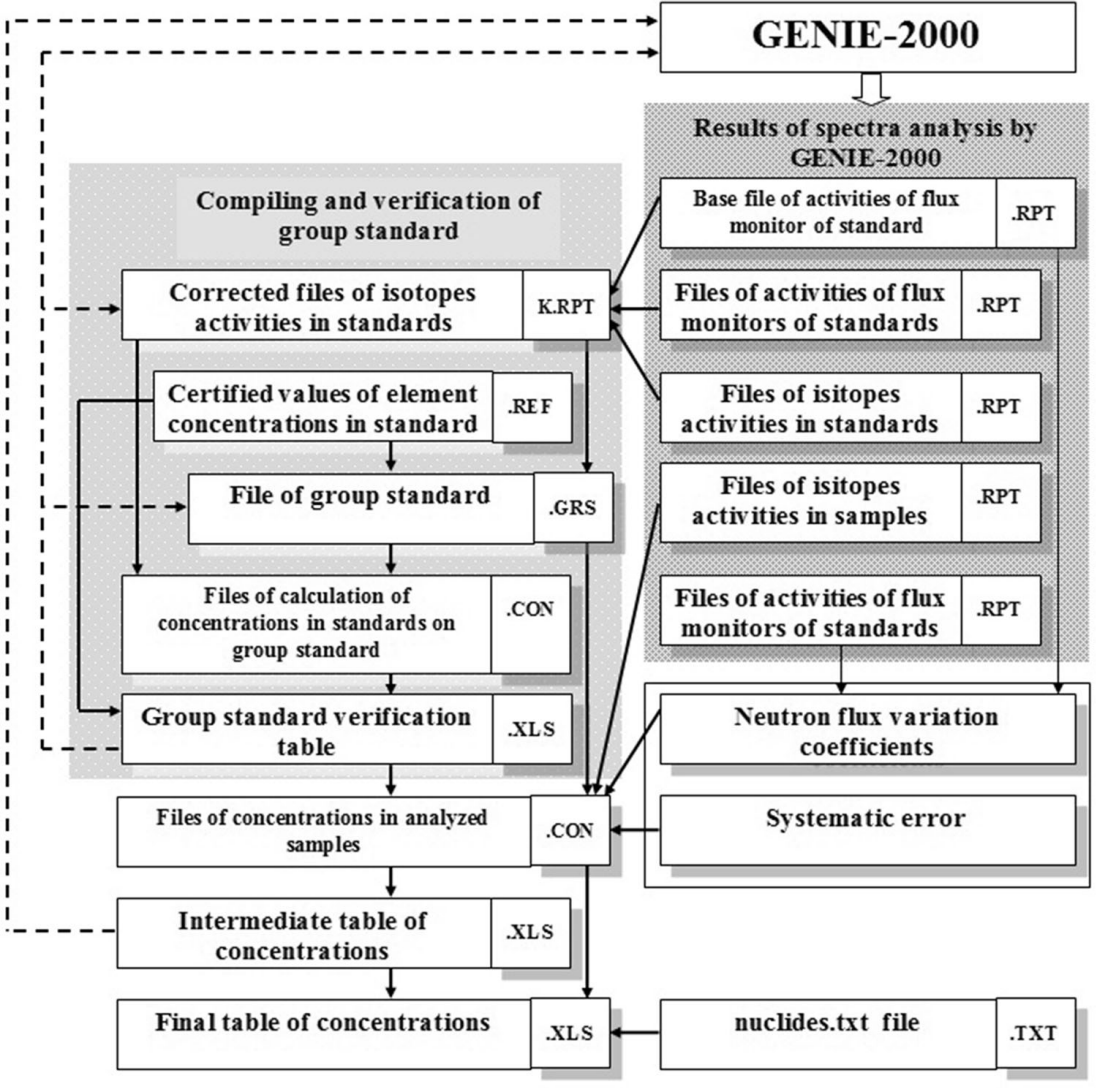
The flowchart of program *Concentration*

Automation of calculation of element concentrations in the samples based on the results of analysis of the spectra of samples, standards, and monitors is achieved by using the program *Concentration*. The flowchart of this program is shown in Fig. 9.

The program uses values of radionuclide activities recorded in output files of the Genie-2000 analysis of spectra of samples, standards, and monitors to calculate the values of element concentrations in the samples. The operation of the program relies on the file *nuclides.txt*. This file contains the list of detected nuclides and the type of measurement files used to detect the nuclides. Because the same nuclides maybe detected after different irradiations and from the data of different measurements, the types of measurement providing the best identification (least uncertainty and maximal sensitivity) are listed in the table *nuclides.txt* file. The file may be edited by adding new nuclides, deleting unneeded ones, and changing the type of measurement. Additionally, files with certified values of element concentration in the standard samples are needed.

Thereby the program *Concentration* allows one to:

- make a correction of activity of isotopes in the samples and standards using the neutron flux monitors;
- make a group standard using several irradiated CRMs;
- check the compiled group standard calculating the concentration of elements in each of the standards through the group standard and making the resulting table where obtained and certified values are compared;
- calculate the concentration of elements in the samples using the group standard;
- compile an intermediate table of results and to check the results by creating graphs based on the elemental concentrations obtained from different measurements;
- compile the final table and to save it into the database.

Intermediate table of results consists of four columns for each radionuclide. It can be stored as MS Excel file (the “xlsx” extension). To verify the quality of data processing, the correlation graphs for ^24^Na–^24^Na, ^122^Sb–^124^Sb, ^141^Ce–^140^La, ^233^Pa–^239^Np are drawn automatically. The first graph is interactive. It is possible to draw any correlation graph at this window.

A final table is created, if there are no errors in the intermediate table. This table is ready to be saved in the NAA database. References [4, 5] gives a detailed description of this program.

**Fig. 10:**
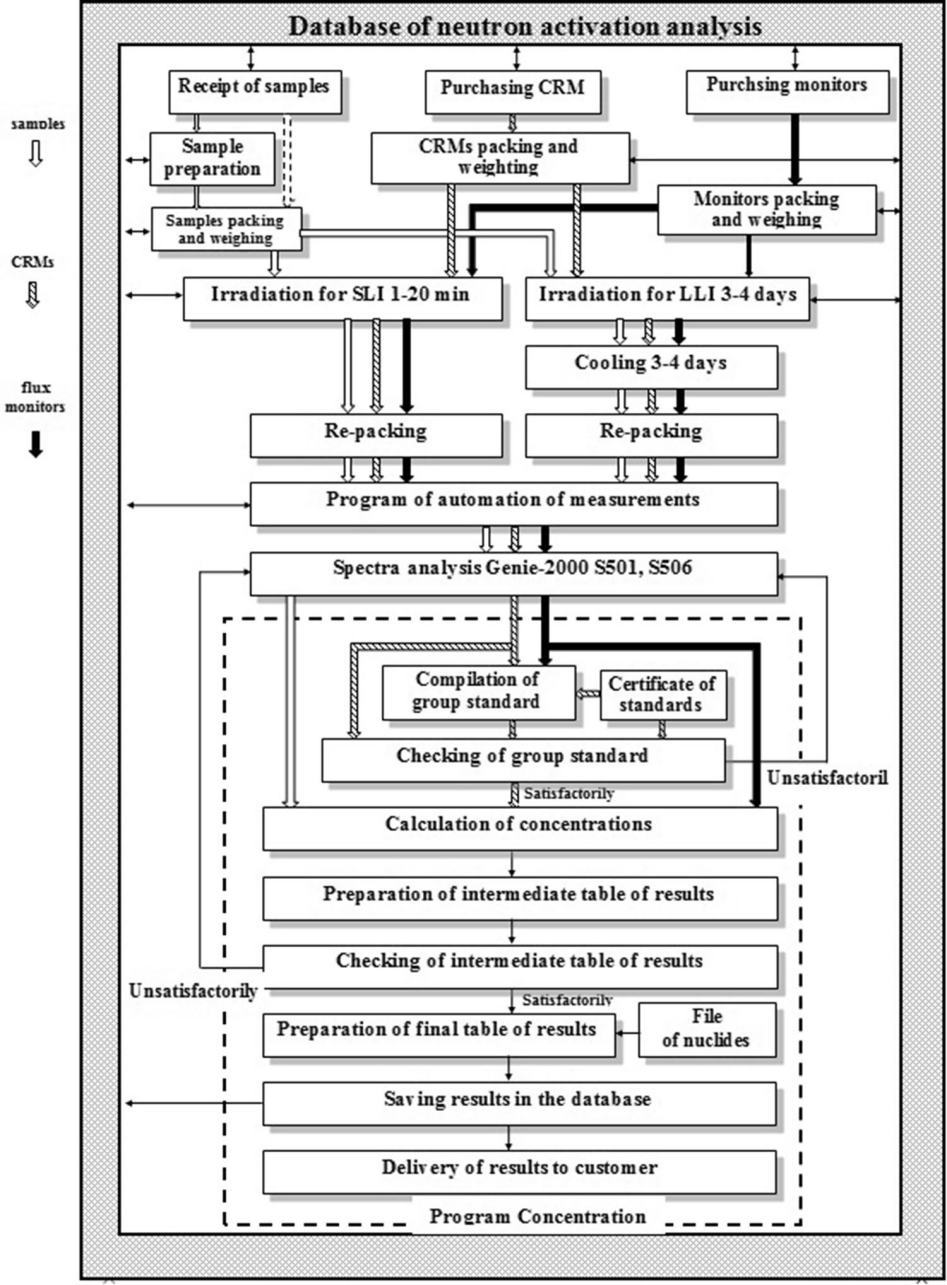
The flowchart of NAA at IBR-2 after modernization

The flowchart of NAA at IBR-2 after modernization is shown in Fig. 10. The main results of automation of NAA are:

- all data about of all stages of analysis are stored in the database;
- the database allows use the electronic document circulation and gives good opportunities of searching, sorting and the analyzing the collected data;
- there is the program and equipment for automation of spectra measurement;
- there is the program for automation of concentration calculation and presentation of final results;
- automation of QC procedures;
- several service programs bringing additional opportunities to automation of NAA.

Automation of sample irradiation remains to be done.

## Conclusions

Software and equipment for the automation of NAA were developed at the FLNP, JINR. Large volumes of data were processed, the efficiency and quality of analysis increased, and the number of human errors performed during NAA decreased. The software developed is in everyday use as a working tool in the Department of NAA and Applied Research at the FLNP, JINR. The implementation of sample changers allows measuring spectra automatically through evenings and nights without presence of operators.

All improvements were achieved by fast and easy access from any PC of our Department to information about all steps of analysis stored at the database; automatic entering the data for spectra analysis; automation of some procedures of QC; automatic measurements of spectra in the out-of-working time; automation of calculation of concentrations; fast and easy statistical analysis of results. Number of samples for determination of short and medium-lived radionuclides increased. However, the number of samples for determination of long-lived radionuclides is limited because of low neutron flux density at irradiation position.

The renewal of pneumatic transport system for sample irradiation is in progress, but full automation of irradiation has many difficulties, especially for long-term irradiation. It is explained, in the first hand, by low neutron flux density of thermal and epithermal neutrons. On the other hand, flux of gamma-rays and fast neutrons in the IBR-2 reactor is very high, that prevents usage of transport tubes, containers and package material from polyethylene because of its low radiation resistance. That is why 80–90 samples are irradiated simultaneously during 3–4 days in aluminum transport containers. After irradiation, the activity of aluminum containers is very high that requests long cooling time before manual repacking of samples into clean polyethylene containers for measurements.

The automation of short irradiation can be achieved with usage of a small inner containers for samples placed in a large transport container. The unit for automatic extraction of small containers from the large ones should be developed. In this case, it will be possible to send samples after irradiation directly to a detector.
